# Inevitable Abortion and Its Treatment

**Published:** 1908-08-01

**Authors:** 


### Obstetrics.

### INEVITABLE ABORTION AND ITS TREATMENT.

The treatment of inevitable abortion in private practice is often a matter of considerable difficulty, and the results are shown by the great amount of chronic illness which follows. A very considerable proportion of patients who have chronic pelvic complaints date their troubles to an abortion, the history of which is one of prolonged bleeding, clearly the result of incomplete uterine involution. The practice of treating inevitable abortion with ergot must be blamed for many of these cases, as the effect of this drug is an abnormal kind of contraction of the uterus, and almost always an incomplete emptying. Products of conception which have been loosened and even detached from the uterine wall are not always expelled if ergot be given, and may become adherent again by organisation of fibrinous exudates or blood-clot. The consequences of this are subinvolution, a flabby œdematous condition of the uterine muscle, which predisposes to displacements and flexions, and a long-continued, congested condition of the organ. If such an organ becomes infected, even by organisms of the mildest virulence, the foundation of a chronic hypertrophic endometritis is laid in addition.

Abortion is said to be inevitable when uterine contractions have succeeded in so far opening up the cervical canal that some part of the contained ovum can be felt projecting through the internal os. As long as the internal os is closed there is always some hope that the attachments of the ovum have not been so completely severed as to lead to the death of the embryo. After the canal and the internal os have opened, and some part of the contents is protruding, it is, as a rule, useless to attempt to stave off abortion any longer. Then it is our duty to empty the uterus completely in the quickest and safest manner possible. No drug can be relied upon to do this successfully, and, in many cases, the uterus seems incapable of it without some extraneous aid. In practice there are two methods which commend themselves as being simple and perfectly satisfactory when properly carried out. The first is the method by which the uterus is stimulated to normal contraction and retraction by plugging the vagina and cervix. The second is digital or instrumental removal of the ovum with or without a general anæsthetic. The first method is that which can be easiest carried out in general practice, when it is often inadvisable to contemplate an operation or to administer an anæsthetic. If vaginal plugging is done properly, it nearly always results in the complete expulsion of the ovum in a few hours. It must, however, be done thoroughly, and the most careful aseptic technique must be observed. It is often stated that it is impossible to carry out aseptic technique in a poor house, with unhygienic surroundings; this is an absolute mistake, and any man can convince himself of the truth of it by a few moments' thought. To carry out an aseptic plugging, nothing is required of the patient except some boiled water; the rest of the necessary materials the surgeon must carry himself, and very little time need be expended in their preparation. The necessary implements are a Sims' speculum, a tenaculum, a pair of sponge forceps, a long dressing forceps, three yards of iodoform gauze, some wool sterilised by soaking in an antiseptic, a pair of boiled rubber gloves, a boiled nail brush and some ether soap. The instruments, gloves, and nail-brush must be boiled at home, and turned out into a towel which has been soaked in one per cent. lysol solution and then wrapped up in a piece of mackintosh; gauze should also be soaked in the lysol solution, as it is not always sterile when dry. These materials can be laid out in the towel in which they are carried, and are ready for immediate use. The vagina must be cleansed before plugging, and the best way to do it is to pour in some ether soap and scrub it vigorously all over the vaginal walls with wool. The cleansing is finished with one per cent. lysol solution. This can be done quite gently, and need not hurt the patient. No douche will cleanse the vagina like this scrubbing process. Then the operator puts on the previously boiled gloves, introduces the speculum, seizes the anterior lip of the cervix with the tenaculum, and inserts the gauze into the cervical canal and lower uterine segment, plugging it fairly tight; lastly, the vagina is systematically filled up with gauze, all in one strip. This plug may be left *in situ* for twelve hours, and, as a rule, the ovum will be found in the vagina, and the uterus empty when it is removed. It need hardly be said that the hands must be scrupulously cleansed or boiled rubber gloves worn before removing the gauze and ovum.

If this method fails, or if it is decided to empty the uterus immediately, the second method may be used. The same cleansing process must be gone through and rubber gloves worn, and in most cases an anæsthetic is necessary. If the internal os admits the finger, the ovum may be at once separated in all directions and then can sometimes be squeezed out of the uterus bimanually. If there is any doubt whether any remains behind, the emptying may be completed by curetting the now partly contracted uterus with a large blunt flushing curette. This, of course, adds to the apparatus required and to the risks of the operation. The real danger of the curette lies in its use whilst the uterus is quite uncontracted, for here there is a danger of perforation. When the uterus has partially contracted the curette may be used without much risk. Nothing further need be done unless the uterus contracts badly and bleeds. Then it is wiser to finish by plugging the *whole* uterine cavity with iodoform gauze for 24 hours. If a little thought be given beforehand as to the easiest way of carrying out the aseptic details of these operations, they will entail a minimum amount of trouble to the surgeon, and can be carried out in the poorest house as well, if not as comfortably, as in a well appointed hospital. The need for an anæsthetic depends much upon the temperament of the patient. The amount of pain involved is not really very great, but one must be able to rely on the patient keeping still. To use the curette by the sense of touch only, without an anæsthetic, is to run a very great risk of perforation and the introduction of sepsis.

